# The Ethiopian telecom industry: gaps and recommendations towards meaningful connectivity and a thriving digital ecosystem

**DOI:** 10.1016/j.heliyon.2021.e08146

**Published:** 2021-10-13

**Authors:** Berhan Oumer Adame

**Affiliations:** Faculty of Electrical and Computer Engineering, Arba Minch Institute of Technology, Arba Minch University, Arba Minch P.O. Box 21, Ethiopia

**Keywords:** Telecommunication, Connectivity, Digital economy, Ethiopia, Sub-Saharan Africa

## Abstract

Ethiopia, the second-most populous country in Africa with 110 million inhabitants, has one of the oldest public telecommunication operators established in 1894. Despite its age, Ethiopian telecommunication remains one of the least developed in the world. According to ITU, in 2019, mobile-cellular subscription (per 100 people) was 39 % and 20 % Internet penetration. As of June 2018, the international transmission speed per Internet user was two kbits/s. Different studies widely acknowledge that no modern economy can be developed short of telecommunication services. It is no wonder that Ethiopia is depicted as one of the weakest economies in the world. This paper identifies the causes for extraordinarily poor telecommunications service in Ethiopia and offers recommendations for near-term improvement. The approach considered includes existing work surveys and document examination, and to this end, the work has relied primarily on secondary data sources. Inexperienced and ineffectual regulatory oversight, absence of facilities-based competition, inadequate interconnection with the neighboring nation's networks, and inability to localize outbound traffics are identified as factors affecting the Ethiopian telecom industry performance. Full liberalization with an effective regulatory body and hosting a sizeable Internet Exchange Points (IXPs) are recommended for better connectivity and a thriving digital ecosystem in Ethiopia.

## 1 Introduction

Founded over a century ago, Ethiopian Telecommunications service provider is Africa's oldest public telecommunication operator. Until 1952, the operator was under the Telephone, Telegraph, and Postal services department. With proclamation No. 131/52 the Imperial Board of Telecommunications (IBTE) was established, in 1952, to expand and provide telecommunications services. Later in 1981, IBTE restructured itself as both a telecom regulator and operator. Then, regulation 10/1996 organized the Ethiopian Telecommunications Corporation (ETC) to operate telecommunication services (Federal Democratic Republic of Ethiopia, 1996a), and in the same year, proclamation 49/1996 established a separate regulatory body, the Ethiopian Telecommunication Agency (ETA) (Federal Democratic Republic of Ethiopia, 1996b). Finally, the Ethiopian government rebranded ETC, the operator, as Ethio Telecom by November 2010.

The first long-distance telephone line was established in 1894, linking Harar with Addis Ababa. Then the inter-urban network started to expand in all directions from the capital Addis Ababa. Telegraph service began in 1906 between Dire Dewa, a city in Ethiopia, to Djibouti (Ethio Telecom, 2021). For international communication, three different Earth Satellite Stations were installed at Sululta from 1979 to 1987. Following the digital exchange launch in 1988, the operator introduced a digital microwave link in 1989 and installed four high-capacity 60 channel domestic satellite stations, in 1994, at Addis Ababa, Gode, Mekele, and Humera (ITU, 2002; Ethio Telecom, 2021). Until 2005, Ethiopia had a 34 Mbps link with Djibouti, a 155 Mbps digital microwave link with Sudan, and a 60-channel old analog link with Kenya. Eritrea's analog link was cut off during the 1990s war and has yet to be reestablished. These circuits connected Ethiopia to more than 20 Southern and Eastern African countries via the PANAFTEL microwave network (ITU, 2002). The remaining international traffic was handled over Intelsat satellite ground station, which had 12,000 channels (ITU, 2002).

After 2005, Ethiopian telecommunication service has undergone a large-scale transformation and modernization. In 2006, the Chinese Import-Export Bank lent a 1.5 billion USD for laying down 10,000 km of national fiber backbone and expansion of 2G coverage to more than 85% of Ethiopian geographical area (Lili, 2009; Workneh, 2014; Adam, 2010; Wall Street Journal, 2014). Ethio Telecom started to deploy 3G for 1.7 million subscribers in 2011 (Wei, 2017). Moreover, it deployed 3.5G and completed 4G in the capital city, Addis Ababa (Wall Street Journal, 2014; Wei, 2017). In 2019/20, Ethio Telecom collected a total of 47.7 Billion ETB (1.4 Billion USD) revenue, which is a 31.4% increment from last year (Ethio Telecom, 2020). According to the operator, this 31.4% revenue rise in a single year was attributed to network expansion and customer experience enhancement (Ethio Telecom, 2020). For instance, massive tariff reductions in fixed broadband services have led to a 135% subscribers increment from the previous year (Ethio Telecom, 2020). And the introduction of newly discounted products has contributed to a 130% increase in data usage and a 19% rise in voice (Ethio Telecom, 2020).

Although Ethio Telecom's revenue improved significantly in 2018 and 2019, the achievement comes from very low base. The revenue remains very low relative to the number of subscribers. Aforementioned can be easily demonstrated by examining other comparative markets. During the 2017/2018 fiscal year operators in Kenya had 45.6 million subscribers with aggregate revenue of 2.48 billion USD (Communication Authority of Kenya, 2018). African largest market Nigeria had 169 million cellular subscribers with total revenue of 7.3 billion USD (National Communication Commission, 2018). However, Ethio Telecom's revenue was 1.38 billion USD with 40 million subscribers (Ethio Telecom, 2019). Therefore, the Nigerian and Kenyan telecom markets are two and half times higher than Ethio Telecom's productivity.

International traffic was one of the leading sources of Ethio Telecom revenue. For instance, in 2002, the international segment constituted 40 % of the total earning (ITU, 2002). Today after the spread of VoIP and the reduction of international settlement rates, international revenue segment is 147.7 Million USD which is 10 % (Ethio Telecom, 2020). Even though it made only 147.7 Million USD foreign currency from its international business, a substantial (318.4 Million USD) loan payment was made – for projects implemented under the Vendor-financing modality (Ethio Telecom, 2020).

Despite recent year's improvement, Ethiopia's telecommunications network remains one of the least developed. According to ITU, mobile-cellular subscription (per 100 people) has reached 39 % in 2019 from 10 % in 2010, and Internet penetration increased from 3 percent of the population in 2010 to 20 percent in 2019 (International Telecommunication Union, 2019; Ethio Telecom, 2020). 2019 Sub Saharan African average mobile-cellular subscription (per 100 people) was 80 % and 28 % Internet penetration. Hence, Ethiopia's 2019 Internet penetration was far below the Sub-Saharan African average and the world average of 53 %. As of June 2018, Ethiopian international transmission speed per Internet user was two kbits/s, while the global average is 76.6 kbits/s and 103.4 kbits/s in neighboring Kenya (ITU, 2018). In international Internet bandwidth utilization, Ethiopia ranks 120 out of 121 countries included in the 2019 Network Readiness Index (NRI) (Dutta and Lanvin, 2019). The ITU information and communication technology development index (IDI) of 2017 ranks Ethiopia at 170th, far below Mali and Rwanda, two landlocked countries with comparable GDP per capita to Ethiopia. The Kenya IDI is almost double. According to the NRI 2019 report, which is a leading global index on impact of ICT, out of 121 economies Ethiopia ranks 116^th^ (Dutta and Lanvin, 2019).

Different studies widely acknowledge that no modern economy can be developed and maintained short of an efficient telecommunications infrastructure and service (World Bank, 2002; Kim et al., 2010; ITU, 2019; Cardona et al., 2013; Vu et al., 2020). It is no wonder that Ethiopia is portrayed as one of the weakest economies of the world. Hence, for Ethiopia to take advantage of telecommunication opportunities and actively participate in the 21st-century digital economy, causes for such a poor telecommunication reality have to be identified.

The Remainder of this paper is organized as follows. Section 2 presents a review of works emphasizing telecommunication and prosperity, while Section 3 describes the methodology. Section 4 discusses the causes for poor Ethiopian connectivity. Recommendations for better connectivity and a thriving digital Ecosystem are presented in Section 5, and concluding remarks are made in the last section.

## 2 Telecommunication and prosperity

A study has shown that developing countries with better connectivity significantly outperforming others during the 1980s and 1990s (World Bank, 2002). According to a World Bank study, every 10 percent increase in broadband penetration in low and middle-income countries results in a 1.38 % GDP increment (Kim et al., 2010). The study also shows broadband having a better growth potential effect than other ICT services (Kim et al., 2010). Another empirical research examined the economic impact of fixed and mobile broadband services in the least developed and landlocked developing countries. This work found that a 10 percent increase in mobile broadband penetration impacts the economy by a 2.5 to 2.8 percent increase in GDP per capita, while a 2.0 percent to 2.3 percent GDP per capita increase for fixed broadband (ITU, 2019).

Moreover, several works have surveyed previous researches to investigate ICT's effect on the economy (Stiroh, 2005; Cardona et al., 2013; Kretschmer, 2012; Stanley et al., 2018). A study reviewed 150 literature and found that majority of them validating a significant positive impact of ICT on a country's economy (Cardona et al., 2013). Another work analyzed 59 econometric studies to assess ICT impact on the economy. The study obtained an overwhelming number of researches confirming a significant positive effect of ICT on economic performance of a country (Stanley et al., 2018). Other works further indicate, impacts of ICT intensifying in the coming decades (Vu et al., 2020).

Even though enormous literatures are supporting the significant positive impact of ICT on a country's economy, there is almost no published research work seeking out why the Ethiopian telecommunication industry is underperforming. Regarding research, the Ethiopian telecom industry is under-covered. However, as discussed earlier, Ethiopia continues to suffer substantial negative consequences from having one of the worst telecommunications services in the world. Therefore, for Ethiopia to take advantage of telecommunication opportunities and actively participate in the 21st-century digital economy, causes for such a poor telecommunication reality have to be identified a-priori, and recommendations must be made for Ethiopia to cope with the current fast pace of change.

Based on a thorough literature review, secondary datasets, the paper proposes an in-depth analysis of the Ethiopian telecom Industry, identifies key weaknesses that could explain its low performance, and draws some recommendations that could support this industry's catch-up. The paper's comprehensiveness, which includes the effect of CDN, IXPs, and the continent's submarine cables investment, adds valuable insight into Ethiopian Telecom industry gaps. In addition, the paper is timely as Ethiopia is preparing to reform this industry. It is also helpful for scholars and policymakers involved in Sub-Saharan Africans' (SSA) digital Ecosystem. Accordingly, this paper primarily makes two contributions. It seeks out causes for Ethiopian poor digital connectivity by undertaking a comprehensive literature survey. And the second contribution is providing recommendations for near-term telecom connectivity improvement in Ethiopia.

## 3 Methodology

The approach considered in this study includes existing work surveys and document examination. To this end, this work relied primarily on secondary sources of data. The unavailability of primary data is the main limitation of this paper. In Ethiopia and many African countries, telecom is regarded as a strategic industry and is matter of national security. Due to this, much of the data is confidential and kept away from the wider public.

This study started with a comprehensive examination of published academic papers on Ethiopian or Sub-Saharan Africa Telecommunication Industry gaps. The search was conducted in four steps. Initially, the search for papers is done by using a set of predefined keywords including “telecommunication”, “Information and Communication Technology”, “telephone”, “Internet”, “mobile phone”, “broadband”, “Submarine cables”, “Data centers”, “Content Development Networks”, “Internet backbone”, “interconnection”, ”liberalization”, “regulation”, “Ethiopia”, “Sub Saharan Africa”, and “Ethio Telecom”. A thorough search is performed in all the major databases of academic papers, including Scopus, Science Direct, Google Scholar, ProQuest and JSTOR.

Secondly, to capture as many relevant citations as possible, wide range of Ethiopian government databases were searched. Various Internet search engines were explored for web pages that might provide references. The electronic searches were supplemented by hand searching for studies in Ethiopian Universities. Furthermore, backward snowballing was used for scanning reference lists of the papers found from the first step to identify additional articles.

Since the above three steps resulted in many citations, lastly, manual screening of all the collected documents is done by using the following selection criteria:

i. The document must be a research article from an SSCI- or Scopus-indexed journal, a thesis published by a reputable University, or a publication by an academically reputable organization such as the World Bank, TeleGeography, ITU and the OECD; and
ii. The document must address status or gaps of the Ethiopian or Sub Saharan Africa telecom industry, otherwise, it must provide global best practices regarding telecommunication industry services, policy, planning, regulation, operation, infrastructure, and Internetworking.

From each document, Ethiopian telecom industry gaps and global best practices are noted, and a list is made along with a description of the document. The description, which is then used for prioritizing documents, includes the outlet, impact factor of the journal, and number of Google Scholar citations the publication received. Identified Ethiopian Telecom industry gaps list is categorized into groups. The following section shows the analysis and discussion.

## 4 Connectivity challenge and policy gap analysis

### 4.1 Inept regulator and telecom monopoly policy

After the 1996 proclamation, Ethiopian telecom operator used to be supervised by the Ministry of Transport and Communications, later called the Ministry of Development Infrastructures (MDI). The Ministry oversees energy, transportation, and postal services besides telecommunications (Federal Democratic Republic of Ethiopia, 2001). It also had control over the regulatory Ethiopian Telecommunication Agency (ETA) (Federal Democratic Republic of Ethiopia, 1996b). It approves the budget and appoints the General Manager for ETA. With the establishment of the Ministry of Communications and Information Technology (MCIT) in 2010, the government reversed the 1996 proclamation and allowed MCIT to absorb the regulator (ETA) as one of its departments with the name Standard and Regulatory Directorate (Federal Democratic Republic of Ethiopia, 2010). Finally, with the closure of MCIT in 2018, a sectoral regulatory and institutional vacuum has occurred.

Until its 2018 closure, the agency had a handful of employees. Considering the experiences of regulators in other similar big countries, such as Nigeria's National Communications Commission (NCC, n.d.) and Egypt's National Telecommunications Regulatory Authority (ENTRA, n.d.), a standard regulatory agency for Ethiopia could have over 500 workforces. Ethiopia has to form ETA (i.e., the regulatory agency) at least with 100 core experts at the start off. The expertise must include communication engineering, economics, and law fields. But in 2002, ETA used to operate with a total of 40 (forty) people. Out of these 40 employees, only a small number (seven or eight) had professional qualifications (ITU, 2002).

Inferring from the above, the importance of having an effective and independent regulator hadn't been acknowledged yet by Ethiopian legislatures. An independent regulator should have maintained a distance from the Ministries (MDI and MCIT), which oversee the incumbent Ethio Telecom. However, ETA functioned as a governmental agency with total political and financial dependence. Moreover, the regulator is organized with under-resourced, unprofessional, and very few workforces lacking high level of expertise on complex issues. Hence, it is fair enough to say that the regulatory agency was not established as an independent and effective agency to license and supervise telecommunication services.

In 1998 Ethiopian government amended the investment proclamation so that part of Ethio Telecom shares is sold to a private partner (Federal Democratic Republic of Ethiopia, 1998). PriceWaterhouseCoopers (PwC), a private consulting company, conducted market valuation and terms of partnership by the end of June 2002. But, up until today telecom industry in Ethiopia is a state-owned monopoly.

Though technological barriers to the telecom sector decreased in the last decades, it remains capital-intensive that depends heavily on an external source of financing (Roseman, 2002). In such a case, private investment has paramount importance as Ethiopia is one of the financially cash-strapped countries. Different studies also showed that liberalized telecom attracting more investment than monopolized ones. Developing countries telecom sector got more private investment, summing 331 billion dollars, than any other sector between 1990 and 2001 (International Chamber of Commerce, 2007). However, Sub-Saharan Africa, which had no private telecom investment until 1993, got only 5% of the global total telecom sector investment by 2001 (International Chamber of Commerce, 2007). Hence, since building a telecom system is expensive and Ethiopia is one of the financially cash-strapped countries, the monopolistic policy led to poor telecom infrastructure and services.

Moreover, the inefficiency of the incumbent operator is one of the predominant causes for poor telecom industry service. During 2017/2018, Kenyan operators had 45.6 million subscribers with aggregate revenue of 2.48 billion USD (Communication Authority of Kenya, 2018). African largest market Nigeria had 169 million cellular subscribers with total revenue of 7.3 billion USD (National Communication Commission, 2018). However, Ethio Telecom's revenue was 1.38 billion USD with 40 million subscribers (Ethio Telecom, 2019). Therefore, the Nigerian and Kenyan telecom markets are two and half times higher than Ethio Telecom's productivity. Such incumbent state-owned telecommunication operator inefficiency can be improved by introducing competition into the telecom market (Osei-Owusu, 2017).

Since liberalization enables better and innovative telecommunication services, it is an essential pre-condition for countries looking to make the most of ICT (Roseman, 2002). Penetration rates in competitive telecom markets have improved at a much faster rate than similar monopolized markets (OECD, 2004).

Furthermore, immediately after instituting a separate regulator, several African telecom markets have witnessed a rapid rise in penetration (OECD, 2004). In 1998, the number of privatized telecommunication firms reached 42 percent from two percent in 1980. Nowadays, Ethiopia, Eritrea, and Cuba are the only countries that have not yet carried out the first-round telecom reforms undertaken by other countries (ITU, 2021). Hence, the absence of facilities-based competition and independent regulatory oversight is the foremost reason for Ethiopia's falling behind its neighbors. If the government aspires to be a regional ICT hub, opening up one of the last remaining state-controlled telecommunications markets and changes in the regulatory environment shall be the first priority.

### 4.2 Telecom-techno-commercial exploitation

The term telecom-techno-commercial exploitation indicates vendors' unfair practices to profit from massive telecom projects. Such gains primarily take advantage of client gaps. The gaps include lack of telecom investment priority list within the country, poor project management, shortage of local technical capacity, and vendor-driven techno-economic analysis and infrastructure deployment.

**Figure 1:**
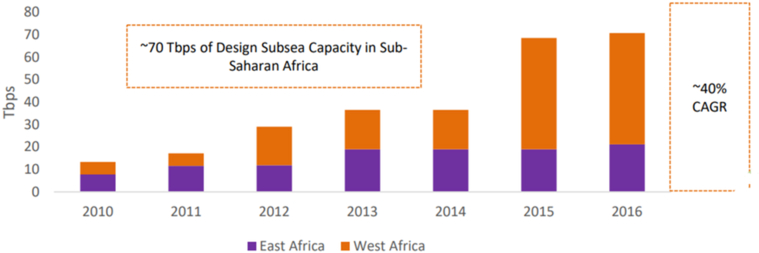
The supply side historical view: Africa Subsea design capacity – 2010–2016 (Reprinted from “The Future of African Bandwidth Markets: African International Capacity Demand, Supply and Economics in an Era of Bandwidth Abundance.”, Xalam Analytics LLC, May 2017, with permission from Xalam Analytics LLC. Retrieved may 06, 2020 (Xalam Analytics Research, 2017)).

**Figure 2:**
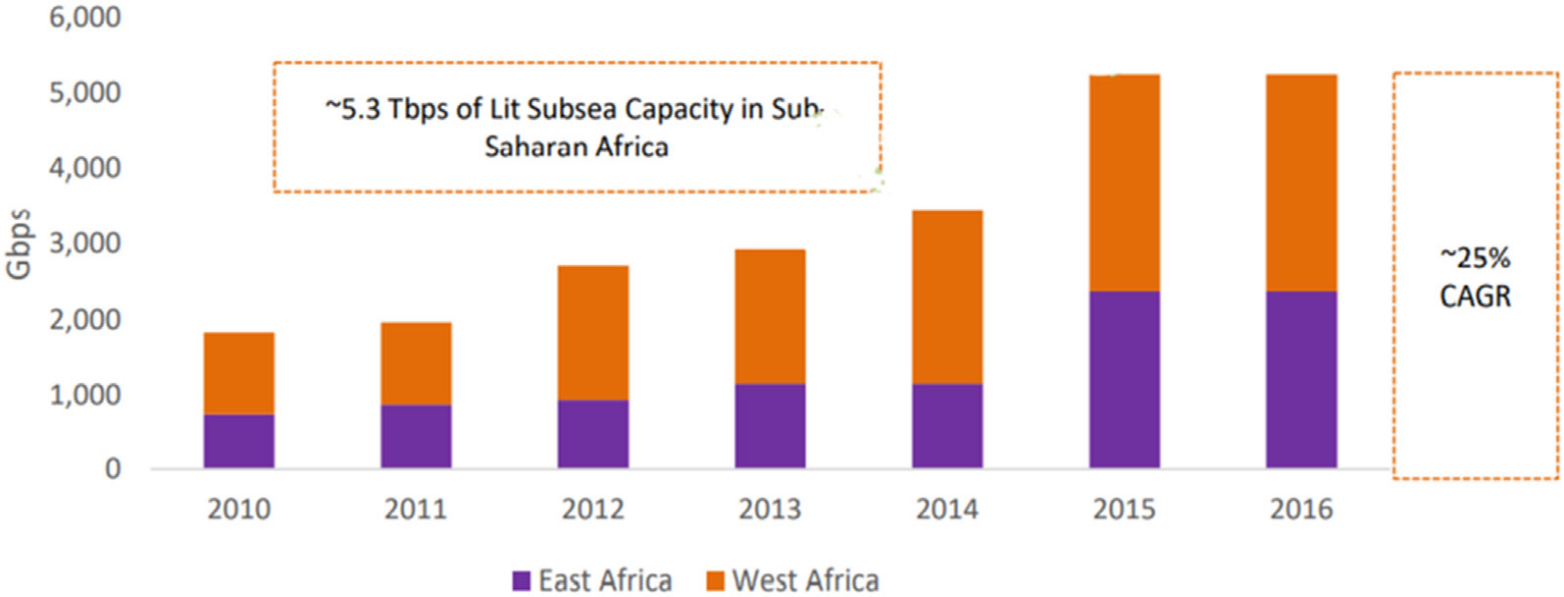
The supply side historical view: Africa subsea lit capacity –2010-2016 (Reprinted from “The Future of African Bandwidth Markets: African International Capacity Demand, Supply and Economics in an Era of Bandwidth Abundance.”, Xalam Analytics LLC, May 2017, with permission from Xalam Analytics LLC. Retrieved may 06, 2020 (Xalam Analytics Research, 2017)).

Referring to how much submarine cable capacity development is going on along the African coastline, one can notice the seriousness of the telecom-techno-commercial exploitation in Africa. Fourteen operational major submarine cables, of which 11 rolled out between 2009 and 2016, providing a total of 70 Tbps in design capacity (Xalam Analytics Research, 2017). However, in 2016, only 5.3 Tbps of lit capacity or barely 7 percent of the design was utilized (Xalam Analytics Research, 2017). The African average annual lit capacity growth was 25% between 2010 and 2016, while the overall capacity supply increasing by nearly doubling every other year since 2011 (Xalam Analytics Research, 2017) (see Figures 1 and 2). The continent's submarine capacity supply increased at a much faster rate than the demand. Such an increase in supply while over 60 Tbps of capacity is waiting for users could lead to bandwidth oversupply (Xalam Analytics Research, 2017).

East Africa's total design capacity was 20 Tbps, whereas just above 2 Tbps was used in 2016 (Xalam Analytics Research, 2017). By December 2018, the used Sub-Saharan Africa international Internet speed reached 5.568 Tbps (Hamilton Research Ltd., 2019). In 2018, 26 submarine cables provided International connectivity to Sub Saharan Africa with a total design capacity of 226.5 Tbps (Hamilton Research Ltd., 2019). The 5.568 Tbps lit capacity is less than 3% of the aggregate design capacity, 226.5 Tbps. There is unambiguous bandwidth oversupply in African Markets. Submarine cable investors are cutting deals with any on-land distributor available.

One could argue, the availability of such large bandwidth is not exploitation evidence but a rather great opportunity for Africans get connected to the Internet. The argument assumes bandwidth buildup leading to a reduced international connectivity price as the competitive play gains momentum, which later will stimulate demand generation. Sub-Saharan Africa's average lit to design ratio of around 3% is very low compared to the Transatlantic route 21.8 %, which is well above the global average of 18 % (Submarine Telecoms Forum, Inc., 2019). A 40 % yearly increase in supply when demand increases at 25 % while already having very low lit to design ratio implies unstimulated consumption. From the bandwidth buildup, neither competitive play gained momentum nor demand generation get stimulated (see Figure 2). Who will consume this bandwidth landed on the African coastline by the different cables remains unanswered, at least as of now. Thus, this bandwidth buildup indicates a possible existence of telecom-techno-commercial exploitation.

Since subsea cables’ lifespan is 25 years, a long-term business strategy is required to stimulate bandwidth consumption. Improving the terrestrial backbone infrastructure and interconnection among individual countries should be the priority for African operators. And most importantly, thorough commercial viability is required during the planning phase of future submarine cable projects.

In 2006, Ethio telecom secured a 1.9 billion USD Chinese Import-Export Bank loan for laying down 10,000 km of national fiber backbone and expansion of 2G coverage to more than 85% of Ethiopian geographical area (Lili, 2009; Workneh, 2014; Adam, 2010). According to a World Bank investigation, the Ethiopian government ignored procurement rules that require competitive bidding when it awarded the contract for ZTE (Geda, 2008; Wall Street Journal, 2014). Competitive bidding is a crucial control that ensures projects are well-planned and clients get the best price. Therefore, the lack of competitive bidding for this contract suggests, Ethio-Telecom could have paid above market price.

Because of the vendor-driven telecom infrastructure deployment, which is common in Africa (Vidal et al., 2012; Smura, 2012), the contract gave ZTE a monopoly on planning, supplying, deploying, and operating telecommunications equipment for several years (Lili, 2009; Wall Street Journal, 2014). The head inspector of ZTE Ethiopia Technology Center, Zhang Jinbao, said: "Operators usually already had plans, so as the supplier, ZTE just had to proceed according to their plans and it would be fine, but there was no plan in Ethiopia, so we had to start from scratch" (Africa China Reporting, 2012). Not only did ZTE plan, supply and deploy but also operated the network for quite some time. Indicating complete dependence on a single equipment supplier and expertise. According to one study (Workneh, 2014), the quality of ZTE telecom products and services has more to do with client competency than with company competency. The marketing and operational strategies of ZTE are usually devised based on an analysis of the customer level of expertise (Workneh, 2014). Therefore, the lack of know-how to make the most out of the deal puts Ethio-Telecom in a disadvantaged position. Ethio-Telecom can potentially get substandard equipment and services (Workneh, 2014).

Using key performance indicators (like time, cost, and quality), a research (Melssie, 2013) evaluated the Fixed Line – Next Generation Network (FL-NGN) project, which is part of the ZTE contract. The evaluation found project deliverables not being met and poor project management practices (Melssie, 2013). Another study (G/Hiwot, 2017) investigated factors influencing the Ethio telecom ICT project implementation and identified a lack of proper project management practice as the root cause (G/Hiwot, 2017). There was also a gap in knowledge transfer from ZTE to Ethio telecom employees (Melssie, 2013). For some solutions, vendor dependency extends for more than three years because of such a gap. Moreover, operation and support cost has required an additional 40% of project cost (G/Hiwot, 2017). (SHEMELIS, 2017) Investigated the Business Support Solution (BSS) project, which is part of the Telecom Expansion Project (TEP) deployed by HUAWEI and ZTE. According to the finding, the BSS project has required an extra budget and time to meet the project objective.

The 2006 ZTE project was awaited for expansion of 2G coverage to more than 85% (Lili, 2009; Workneh, 2014; Adam, 2010). However, after the ZTE monopoly in July 2013, another project with a 1.6 billion USD cost was awarded to ZTE and Huawei Technologies for TEP. The TEP project promised to increase 2G coverage from 64% to 90%, improve 3G network coverage in rural areas and introduce LTE in Addis Ababa (Wall Street Journal, 2014). However, currently, Ethio Telecom has around 85 percent 2G coverage, 66 percent with 3G, and 4 percent with 4G (The World Bank, 2019). It owns 7,100 cellular towers mainly connected by microwave links rather than fiber optics (The World Bank, 2019). Ethiopia remained at the bottom of major global indices such as the Network Readiness Index (NRI), the International Telecommunications Union ICT Development Index, and the Alliance for Affordable Internet (A4AI) Affordability Drivers Index. Ethiopia was ranked 116th out of 121 countries included in the 2019 NRI and 60th out of sixty countries surveyed in the 2018 A4AI Affordability Drivers Index (Alliance for Affordable Internet, 2018).

Although it is difficult to infer the extent of possible telecom-techno commercial exploitation without auditing the entire contract, in an interview with the wall street journal, the chief executive of ZTE's Ethiopian branch, Jia Chen, admits that the network service has been uneven. He acknowledges the existence of poor network coverage within cities including Addis Ababa, frequent call drops, poor voice quality, and call Congestion (Wall Street Journal, 2014). Despite the different outcomes, both the first and the second deals together surpassing 3 billion USD, Ethiopian telecom should have been in much better condition if there hadn't been serious telecom-techno-commercial exploitation.

### 4.3 Lack of interconnection among countries within the region

Lack of network interconnections among African countries is common, especially those in the interior. Data traffic destined to neighboring countries is often shipped overseas to return to Africa. Most African cross-border Internet traffic still exchanges in Europe and North America (Fanou et al., 2017). Ethiopia upstreams entirely through overseas providers. The country's 70% of paths go through Europe and 30% via North America (Formoso et al., 2018). According to a study, on average, 75% of packets from Africa were destined to African traversed inter-continental links in Europe (like London, Marseille, and Amsterdam) (Chavula et al., 2015).

Independent analysis supported by African Union has also shown that Africa pays more than $600 million annually to off-continent Internet transit providers, particularly for intra-country and intra-African traffic exchange routed outside the continent through costly transit links (African Union (AU), 2009). The cost is growing as time goes by (Internet Society, 2012). Such cost not only contributes to high end-user access price but also ships precious financial resources overseas.

An Autonomous System path (AS path) is a list of all Autonomous Systems (ASes) that a particular route passes through to arrive at a destination. Border Gateway Protocol (BGP) router adds neighbor AS number to the front of the AS path when it receives an update from a neighbor AS. Therefore, the path gets longer as the route announcement circulates from AS to AS. A study (Fanou et al., 2017) showed AS path length distribution for all the intra-African routes. According to this study, the East African area has a longer AS path, 6 ASes on average (Fanou et al., 2017).

A striking result also observed in Round Trip Times (RTTs). Around RTTs > 2s were recorded in paths, especially in ISPs between East African and West African (Fanou et al., 2017). Moreover, African intra-country latencies average at 78ms, higher than North America 45 ms, and 30 ms of Europe (IP latency statistics, 2017). Many African countries suffer from severe tromboning effects. This phenomenon affects performance such as latency and reliability (Kini, 2014). Due to this, in many cases, it is faster to get to North American or European networks than to reach African from another African network (Formoso et al., 2018).

In February 2014, out of the global 435 IXPs, Africa had only 5% and increased to 7.5% of the global 491 IXPs available by July 2016 (Packet Clearing House, 2016). Furthermore, almost all IXPs in Africa are small and isolated (Augustin and Willinger, 2009). Africa's largest IXPs are in South Africa (having a maximum of around 160 members), and 16 is the average number of African IXP members. Except for South African, the scale of African IXPs is insignificant compared to European or North American IXPs that are peering 500 ASes (Galperin, 2013). For instance, Ireland's Internet Neutral Exchange Association, an IXP functioning in one of the smallest states in Europe, maintains 127 members with 200 Gb/s traffic. It is 40 times larger than one of the largest IXPs in East Africa, the Uganda Internet eXchange Point with traffic of 5 Gb/s maintaining 26 members (Briain, 2019). IXPs in Africa and the East African sub-region are insignificant in size and number.

Ethiopia upstreams entirely through overseas providers. 70% of paths go through Europe and 30% via North America (Formoso et al., 2018). Considering the latency penalty of such long-haul circuitous links (Ethiopia → North America is at 144 ms and Ethiopia → Europe is at 354 ms), Ethiopia has high RTT values (Formoso et al., 2018). Due to such use of transit providers that route traffic through N. America and Europe, the network suffers from severe tromboning effects (Formoso et al., 2018). Ethiopia needs to address these issues through more local peering and interconnection across the entire African continent (Formoso et al., 2018).

And thus, operators and stakeholders need to intensify their efforts to localize intra-African traffic through peering. Regional policymakers and regulators need to address one of the leading African Internet development obstacles, the lack of interconnection among countries.

### 4.4 Poor traffic localization and lack of CDNs

Africa contains only 35% of all Google caches (Fanou et al., 2016). Most of these caches are in South Africa, Egypt, Mauritius, Kenya, and Nigeria. There is no single Google cache in most parts of central African countries. Though Google operates from some parts of Africa, other content providers, including Facebook, used to remain outside the continent (Huang et al., 2013). For instance, amazon.com serves Africa from the United States (via AS16509 Amazon and AS46475 Limestone Networks), yahoo.com serves Africa from the Great Britain and United States (both hosted in Yahoo's AS) (Fanou et al., 2016). Even local websites host outside of the continent. Out of 18 regional websites, only five front-ends are hosted locally, particularly in South Africa (Fanou et al., 2016). Hence, compared to other content providers, Google is considerably more established in Africa. All of the above showing the existence of poor traffic localization within the region.

Globally, today Internet traffic has changed from mainly being static text and picture-based content to video. Cisco predicted 82% of all the consumer Internet traffic in 2020 to be video (Cisco, 2019). Moreover, it predicted 70% of all the Internet video traffic in 2020 to be carried by CDNs (Cisco, 2019). In light of this, CDNs are hugely investing in digital infrastructure. Yearly, they spent around 75 billion USD for submarine cables and data center facilities during 2014–17, double the average of 2011–13 (Analysis Mason, 2018). To reach many more localized cloud facilities, CDNs extend their networks by spending on data center diversification.

**Figure 3:**
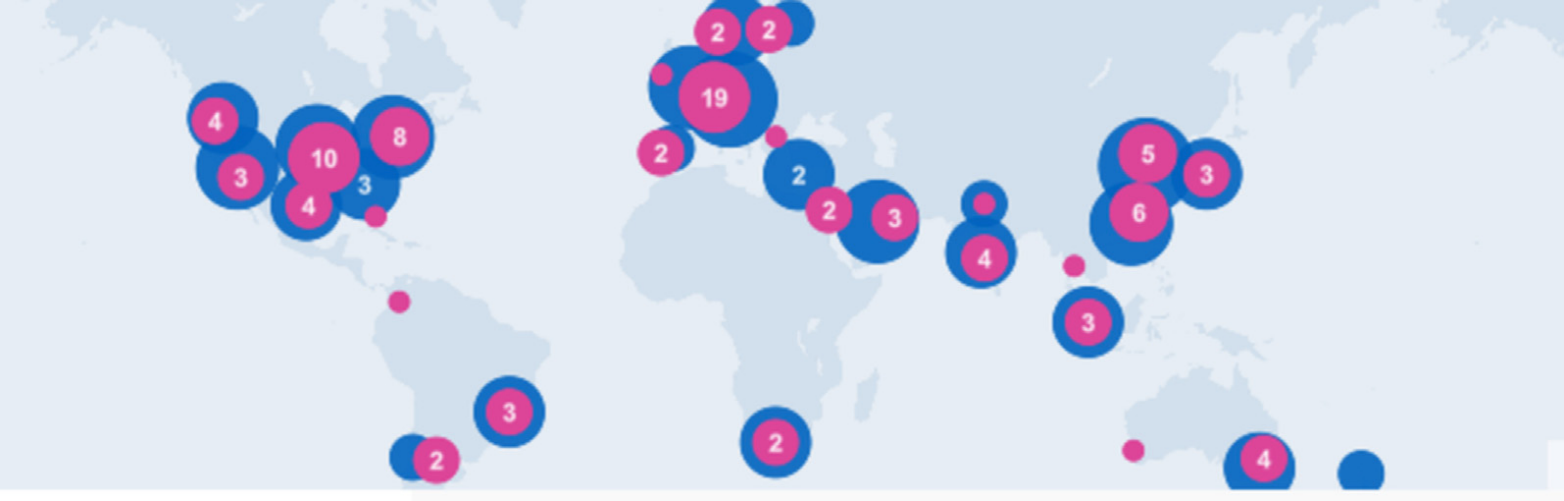
Content delivery networks data centers (Reprinted from “The Cloud Infrastructure Map”, © 2021 PriMetrica, Inc. with permission from TeleGeography. [available at: https://www.cloudinfrastructuremap.com/#/] accessed on December 2020).

TeleGeography mapped regions with Google, Amazon Web Services, IBM Cloud, Microsoft Azure, Alibaba Cloud, and Oracle cloud platform (TeleGeography, 2020). According to TeleGeography, there are 122 data centers in Asia, 98 in the US and Canada, together 67 % of the global cloud data centers, while only 2 CDN data centers are available in Africa, specifically in South Africa (TeleGeography, 2020) (see Figure 3).

**Figure 4:**
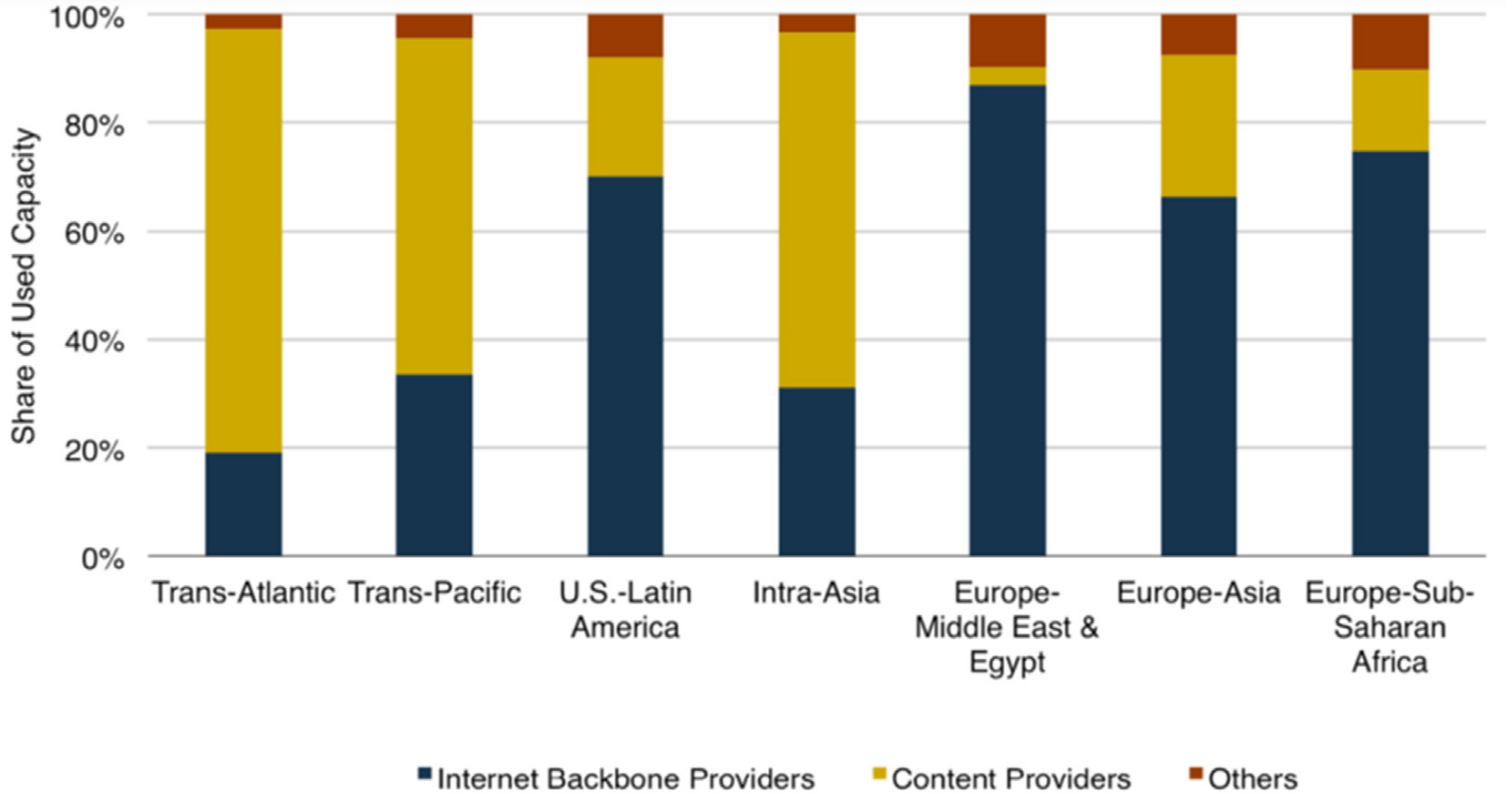
2017 Whose traffic in those pipes (Reprinted from “International Bandwidth and Pricing Trend”, TeleGeography with permission from TeleGeography [available at: https://www.afpif.org/wp-content/uploads/2018/08/01-International-Internet-Bandwidth-and-Pricing-Trends-in-Africa-%E2%80%93-Patrick-Christian-Telegeography.pdf [accessed on September 2020].

Since CDN data centers need to share content and provide back-ups and redundancy, they lease or invest in submarine optical fiber networks. Globally, the total international capacity deployed by CDNs jumped to 962 Tbps in 2019, and by 2017, CDNs had topped Internet backbone providers as the biggest consumers (Mauldin, 2020). However, unlike other regions, African connectivity to Europe is mainly provided by Internet backbone providers (see Figure 4).

Therefore, the lack of CDN data centers and traditional Internet carriers dominated international connectivity indicates the unavailability of enough CDN networks within the continent.

### 4.5 Inadequate terrestrial optical network

Ethio-Telecom has around 22,000 km long-distance fiber network organized in 13 rings. Its 7,100 cellular towers are mainly connected by microwave rather than an optical link (The World Bank, 2019). If cell towers are not connected by optical fiber (and instead rely on satellite or microwave links), capacity will be lower. Users likely are restricted to 2.5G system speed, not broadband. For 3G, 4G, 5G and beyond, optical network backhaul is vital. However, most backbone infrastructure in Ethiopia is a low-capacity wireless network that bases on microwave links.

**Figure 5:**
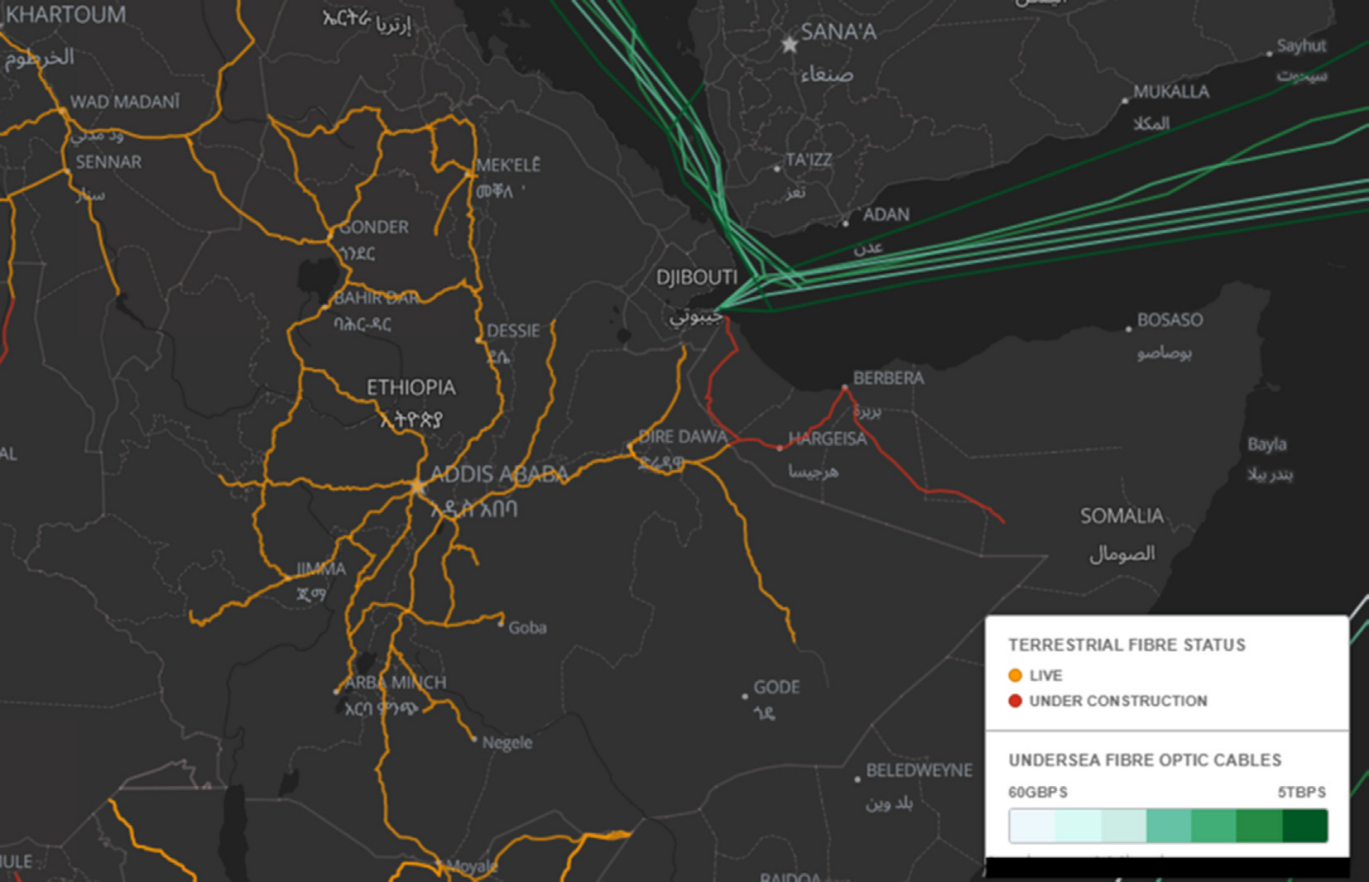
Ethiopia terrestrial fiber network map (Reprinted under the Creative Commons Attribution 4.0 International (CC-BY-4.0) from “African Undersea and Terrestrial Fibre Optic Cables”, Network Startup Resource Center (NSRC), available at: https://afterfibre.nsrc.org/[accessed on: 12/29/2020]).

Relatively redundant parallel fiber networks are laid around the central and northern parts of Ethiopia (see Figure 5). This redundancy increases the network resilience in case of a cable cut. However, much of the east, west Ethiopia and the Amhara region remains fragmented. Insufficient optical network covers these areas while population density is much higher. Furthermore, Ethiopia being the most populous landlocked country, the eastern and southern terrestrial network connecting Ethiopia to the Djibouti and Kenya submarine cable landing sites should have been highly redundant. The redundancy increases network reliability and provides quick in-system restoration when cable cut occurs. However, Ethiopia has a single route towards Djibouti and another one path to Kenya (see Figure 5). There are no multiple loops means no quick in-system restoration, affecting link reliability.

### 4.6 Absence of data-driven decisions

Commercial investment, consumer choice, and policy decisions should base on timely and accurate data. Yet today considerable gap is noticed in the availability of such data. Unavailability of accurate and timely data is quite common, not only in developing but also in developed countries. Microsoft's estimation of Americans lacking broadband access turns up to be much greater than the US Federal Communications Commission estimate, questioning the whole statistical reliability (Smith, 2018).

Ethiopia needs to explore statistical data at the national level to visually represent a more detailed analysis of the needs and connectivity gaps within the country. The availability and the more disclosure of terrestrial maps and points of presence, mobile network tower locations and coverage maps, capacity utilization factor, and pricing data for the middle mile and core infrastructure connectivity, the more targeted policy and investment can be to help fill in the gap.

### 4.7 Prohibitive international bandwidth pricing

2018 International transmission speed per Internet user was 103.4 kbits/s in Kenya (ITU, 2018) while two kbits/s in Ethiopia (ITU, 2018). In International Internet bandwidth utilization, Ethiopia ranks 120 out of 121 countries included in the 2019 Network Readiness Index (NRI) (Dutta and Lanvin, 2019). Ethiopia has a huge gap in international bandwidth utilization, and the demand for faster connectivity shall be satisfied in no time.

**Figure 6:**
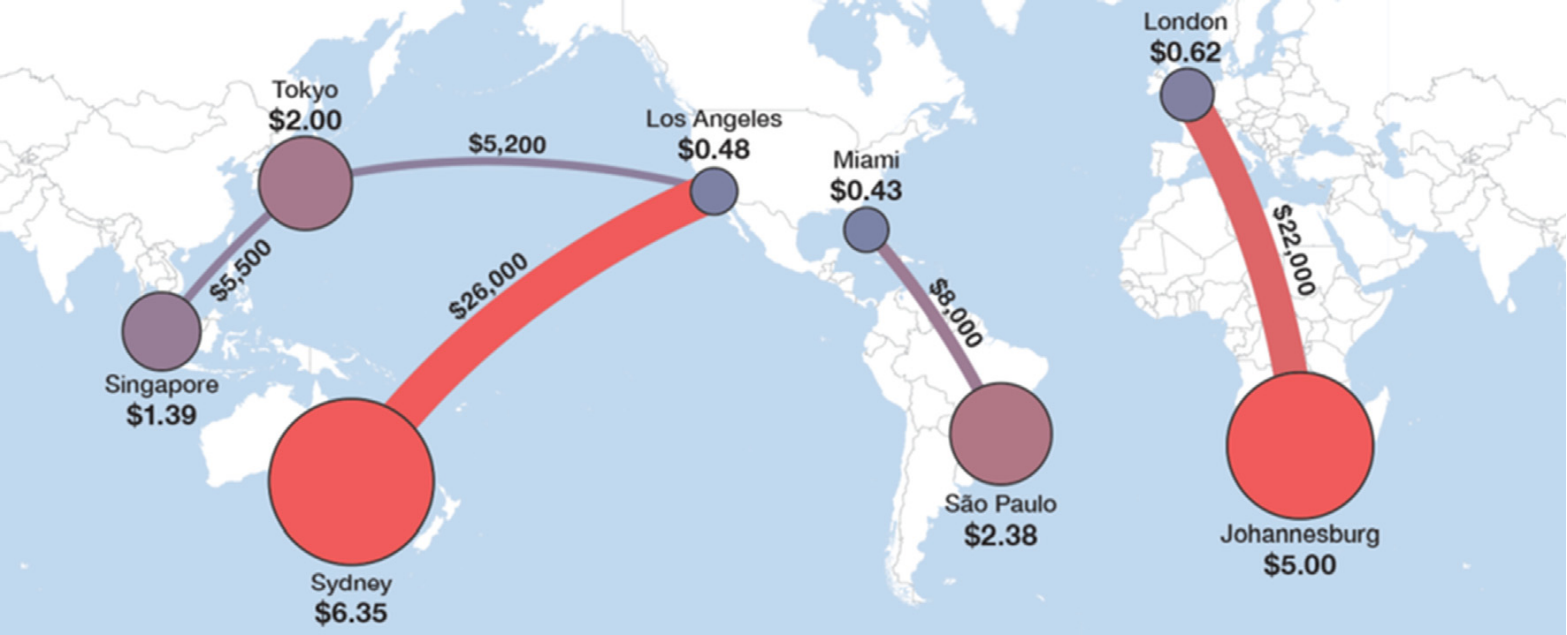
International 10G Wavelength MRC, 10G IPT Port MRC,2018 bandwidth pricing (Reprinted from “International Bandwidth and Pricing Trend”, TeleGeography with permission from TeleGeography [available at: https://www.afpif.org/wp-content/uploads/2018/08/01-International-Internet-Bandwidth-and-Pricing-Trends-in-Africa-%E2%80%93-Patrick-Christian-Telegeography.pdf [accessed on September 2020].

By December 2018, Africa's total used international Internet speed reached 10.962 Tbps, with Sub-Saharan Africa consumption reaching 5.568 Tbps (Hamilton Research Ltd., 2019). In 2018, 26 submarine cables provided International connectivity to Sub Saharan Africa with a total design capacity of 226.5 Tbps (Hamilton Research Ltd., 2019). The 5.568 Tbps lit capacity is less than 3% of the aggregate design capacity, 226.5 Tbps. This enormous supply of international connectivity should have led to a reduced connectivity price due to expected growing competition. Based on weighted median pricing, in 2018, 10G IP transit price was $0.6/meg in London while $5/meg in Johannesburg, nine times higher (see Figure 6). 2017 weighted median 10 Gbps lease price on the Johannesburg – London and Nairobi - London routes was 34,000 and 95,000 USD, respectively (Boudreau, 2020; Christian, 2018). Africa pays the highest price for international bandwidth.

Such pricing has a considerable impact on Internet pricing and puts African operators under pressure. Unless cheap international connectivity is achieved, no matter what new investment in other parts of the telecom value chain is done, a significant price reduction is very unlikely to occur for end-users (Jensen, 2009).

## 5 Recommendations for better connectivity and a thriving digital ecosystem

Ethiopia is characterized by a weak economy and is one of the countries with the least developed telecommunications infrastructure. If Ethiopia has to cope with the current fast pace of change, this paper recommends the following.

### 5.1 Promote telecom market liberalization

The absence of facilities-based competition and independent regulatory capacity is the foremost reason for Ethiopia's falling behind its neighbors. If the government aspires to be a regional ICT hub, opening up one of the last remaining state-controlled telecommunication markets and changes in the regulatory environment shall be the priority. As liberalization enables more, better, and innovative communication services, it is an essential pre-condition for countries looking to make the most of ICT (Roseman, 2002).

Inefficiency of the incumbent state-owned telecommunication operator was the first and foremost reason for introducing telecommunication sector competition in Sub-Saharan Africa. The competition started to take place in the 1990s, which eventually led to the adoption of full telecommunication sector liberalization (Tidiane, 1995; Osei-Owusu, 2017). Not only competition leads to incumbent efficiency but also has a spillover effect on other companies the incumbent deals with.

Furthermore, liberalization attracts new investment and increases the rollout of entirely new telecom services. Countries that made liberalization commitments a priority also have a higher fixed-line and mobile penetration than a monopoly (Roseman, 2002; World Bank, 2003; OECD, 2004). Thanks to more aggressive competition eased by supportive regulatory policies, Zambia witnessed a sharp increase in Internet users within three months, up by 25% of the previous total (IT Web Africa, 2019). Promotion of competitive digital services, particularly for low-income consumers, allowed India's number of broadband subscribers to reach 553.54 million by April 30 2019 (TRAI, 2019). Moreover, competitive telecom markets have higher telecom sector revenue than those with monopolies (Roseman, 2002; World Bank, 2003; OECD, 2004).

Therefore, considering the liberalization overall benefit to the economy and the society as a whole, the Ethiopian government must take a long-term and strategic view of liberalization.

### 5.2 Establishing an independent and effective regulatory agency

An independent and effective regulator is mandatory for successfully transforming a monopolistic telecom industry into a competitive one. Viable competition is unlikely to arise and develop without it. Countries' experience showed that unavailability of efficient regulatory oversight leading to a substitution of a public monopoly by a private one (S & A, 2015). It is mandatory to empower the regulator and allocate resources in line with the national telecommunication industry. Moreover, it must consist of an appropriate mix of economics, network technology, and law fields of expertise. Therefore,

• Providing a reliable financial source for the agency,
• Establishing an independent board of directors, and
• Running the regulator with a clear legal mandate are crucial for the independent and effective functioning of the regulator.

### 5.3 Mobilize investment from various stakeholders

Ethiopia, a country of more than 110 million inhabitants, had only 20 percent Internet penetration by the end of 2019 (International Telecommunication Union, 2019). According to an expert 2016 cost estimate, ensuring universal connectivity requires 300 USD per person (International Telecommunication Union and Broadband Commission, 2016). The World Economic Forum analysis on East Africa set a 115 USD investment per person price tag for ensuring universal connectivity (World Economic Forum, 2017). According to a report by the World Bank Group, achieving universal and better quality Internet for Africa by 2030 requires an investment of US $100 billion. Therefore, Ethiopia requires massive telecom infrastructure investment due to the existing large-scale limits of network coverage, middle-mile and first-mile connectivity.

Such a substantial infrastructure and funding gap requires effort from all stakeholders. It demands a broader mobilization of potential sources of investment at the country's disposal. With this work, some means of filling the investment gap are discussed next.

#### 5.3.1 Utilize universal service funds and universal service obligations

One way of funding universal access policy is via universal service funds and universal service obligations. Many countries collect universal service funds through a compulsory contribution from service providers and network operators. These funds can then use for expanding telecom networks to areas that may not be getting commercial investment. The 2013 ITU report listed 69 universal service funds (16 in the Asia Pacific, 22 in Africa, 7 in the Arab States, 8 in Europe/CIS, and 16 in the Americas) (ITU, 2013).

Many countries are also incorporating universal service obligations, a condition that everyone can access telecom services somewhere in a public place. Furthermore, it includes providing public access Internet facilities to underserved areas (ICB4PAC, 2013; Sulistyo, 2018). In February 2000, the Estonian legislatures added access to the Internet to universal service list of the country. The parliament enacted article 5 subsection (1) (Psaila, 2011) “Internet service which universally available to all subscribers regardless of their geographical location, at a uniform price.” And many more countries are indicating that Internet access is a legal right (Psaila, 2011). Hence, the Ethiopian government must consider using universal service funds and universal service obligations to narrow the digital divide.

#### 5.3.2 Public fund for underserved areas

Often private investment fails to provide network connectivity to lower-income and less densely populated areas. The reason for this is the economic incentive. Private investment in infrastructure is very unlikely, especially delivering high-speed connectivity to places with low returns on investment. In such a case, there shall be a role for public financing. The social returns from providing connectivity to disadvantaged communities are much greater than the financial returns for a government. The Indonesian government rolled out a national undersea optical fiber cable network while the government of Brazil is using satellites to connect remote regions (Pathways for Prosperity Commission, 2018). Ugandan Communications Commission collaborating with the operator, MTN, provides high-speed data to two rural communities using the Intelsat satellite system (ITSO, 2018). Already governments are connecting their underserved citizens effectively by allocating public funds (Pathways for Prosperity Commission, 2018).

#### 5.3.3 Attracting investment from CDNs

Globally, CDNs have become a significant new source of investment. Around 75 billion USD is spent yearly on submarine cables and data center facilities during 2014–17, double the average of 2011–13 (Analysis Mason, 2018). However, there is little CDN investment within the African continent. Out of the top 6 cloud service providers, only Amazon Web Services and Microsoft Azure data centers are available in Africa, South Africa Cape Town (Amazon Web Services, n.d.; TeleGeography, 2020). Moreover, the continent's connectivity to Europe is mainly provided by Internet backbone providers, unlike other regions CDN based connectivity.

Though top CDN operators have a weak presence in Africa, Eastern African countries are attracting some CDN investments. Cloudflare has eight PoPs in the continent, one in East Africa (Cloudflare, 2015), while Facebook has node appliances in Rwanda, Uganda, Kenya and Burundi. Akamai with at least five CDNs in Kenya as well as CDNs in Tanzania, Uganda and Rwanda. Netflix CDNs in Kenya and Rwanda (East Africa Communications Organisation, 2017) and Google edge PoPs in Kampala and Mombasa (Google, n.d.). Comparing with Ethiopia, the other Eastern Africa countries are attracting more CDN investment.

The population of Ethiopia can offer large national economies of scale to content providers. Therefore, the Ethiopian government and policymakers need to devise policies that incentivize investments from data centers and CDN operators. The Ethiopian government and policymakers need to do more to attract investment from CDN operators.

### 5.4 Tapping into Ethiopia potential to host large IXP

Utilizing the IXP network model is key to reduce the cost of Internet and improve network performance such as latency, packet loss, and hop count (Ahmed et al., 2017; Chiu et al., 2015; Briain, 2019). Achieving greater regional interconnection and traffic aggregation at key hubs will let African providers to negotiate peering terms with large international transit providers (Analysys Mason, 2010). Negotiated peering terms allow reduction of international IP traffic wholesale price (Analysys Mason, 2010). A study surveyed more than 100 network operators and peering coordinators to look into the motives of establishing new interconnection agreements. The study found reducing latency, decreasing cost, and improving resilience as the most common reasons for forming new interconnection agreements (Marcos et al., 2020).

Although the IXP network model is imperative, African operators are not benefiting from it. In 2014, Africa had only 5% of the global 435 IXPs and increased to 7.5% of the total 491 IXPs by July 2016 (Packet Clearing House, 2016). Furthermore, almost all IXPs in Africa are small and isolated (Augustin and Willinger, 2009). ISPs operating outside of Africa still dominates intra-African communications interdomain routing (Fanouu and Aben, 2015; Fanou et al., 2017). And thus, operators and stakeholders need to intensify their efforts to localize intra-African traffic through peering.

Ethiopia, Africa's second most populous country, has one of the lowest Internet penetration in 2019, about 20 percent (International Telecommunication Union, 2019). Large potential users are expecting to connect to the Internet, especially the minute prices decrease and QoS improves (Crowcroft and Sathiaseelan, 2015). In addition to the local market, Ethiopia has positioned itself at a strategic location on the continent, centered between Africa, Europe, and Asia. Due to this, Ethiopia has the potential to host large IXPs.

Considering the strategic location of the country and the awaiting large potential users, the Ethiopian government must consider issuing separate licenses for IXPs and international gateway providers. However, it is unlikely to attract such investment to Ethiopia, provided that broadband investment tends to follow regional trade and economic activities. Hence, the government shall put various incentives to catalyze and set off the IXP potential of Ethiopia.

### 5.5 Improve citizens digital literacy

Ethiopia must also invest in digital skills in addition to digital infrastructure investment. The 2018 GSMA Intelligence Consumer Survey showed that one of the main obstacles preventing telecom users from utilizing the mobile Internet is a lack of digital skills and literacy (GSMA, 2019). Hence, Ethiopia to get the utmost benefit from a fully digitalized society, citizens must become digitally literate. Improving digital skills shall be taken as part of the effort to increase connectivity. Well-developed digital literacy lets citizens appreciate the value of the Internet and enhance their Internet know-how. Moreover, it helps them in developing digital resilience.

### 5.6 Building local technical capacity

Local technical capacity must be developed to avoid vendor-driven techno-economic analysis. Ethiopian technocrats & economists can undertake a joint review of all existing projects. In addition, unnecessary future projects can be scrapped, inflated projects can be re-negotiated, and future projects guideline can be created by developing local technical capacity.

Ethiopia also needs to explore statistical data at the national level to visually represent a more detailed analysis of the needs and connectivity gaps within the country. Building local technical capacity in identifying the main broadband infrastructure gaps with a priority list for investment and government subsidy is crucial. Preparing universal service obligation directives and establishing innovative financing mechanisms also demand local expertise. Moreover, the country needs advanced telecom skills to develop applications and services, define priorities for the radio frequency spectrum allocation, and map network coverage.

Such skills benefit private sector operators, government, and development partners as they seek to bring more Ethiopian online. One way of building such capacity needs tightening of bid eligibility to favor local enterprises and foreign companies that have incorporated means of technology transfer on their bid document. In doing so, the country can reduce foreign technical expertise dependency.

### 5.7 Encourage local content development and local hosting

Many studies revealed that one of the causes for the stunted African Internet adoption is the lack of locally relevant content (Fanou et al., 2016; Quast, 2016). Local content, especially content in local languages, will draw new users to the Internet. So, the government or operators should invest in locally relevant content by running various initiatives. Such initiatives include organizing competitions for local IT start-ups, investing in incubation centers, providing open API platforms, supporting local e- Businesses and directly investing in local content companies.

Mainly, Europe and the United States provide content to Africa. Moreover, the most well-known websites of Africa are hosted overseas (Fanou et al., 2016). Hence, the government shall strategically host content at local data centers as part of the effort to develop the Ethiopian Internet Ecosystem. Initiatives to promote and run various e-governance platform is crucial in this regard.

## 6 Conclusion

This paper identifies the causes for extraordinarily poor telecommunications service in Ethiopia and offers recommendations for near-term improvement. In Ethiopia, the state owns telecommunication infrastructure and services. This monopolistic policy led to under-investment of the telecom sector and poor telecom services. Moreover, there is no independent and efficient regulatory agency; the regulator has few professional staff. Therefore, the absence of facilities-based competition coupled with inexperienced and ineffectual regulatory oversight is the leading cause for Ethiopian poor telecommunication service.

As it is common in many African countries, vendor-driven telecom infrastructure deployment, poor project management, and a shortage of local technical capacity are causes for telecom-techno-commercial exploitation. Due to insignificant interconnection among African countries, 75% of packets from Africa destined to African traverses inter-continental links in Europe. A striking result also observed in RTTs, around RTTs > 2s, were recorded in ISPs between East African and West African countries. Moreover, poor traffic localization, lack of enough CDNs within the region, inadequate terrestrial optical networks, and decision-makers drawing on inaccurate and untimely data are identified as factors affecting Ethiopian connectivity. Unless cheap international bandwidth is achieved, no matter what new investment in other parts of the telecom value chain is done, a significant price reduction is very unlikely to occur for end-users. Therefore, costly international bandwidth pricing is identified as another cause for the poor performance.

This study has also outlined several recommendations on the entire value chain. First and foremost, Ethiopian government should take a long-term and strategic view of the overall benefits of liberalization. The legislature should push forward on opening up the last remaining telecom market of the world. From various country's experiences, it is also noted that independent and efficient regulatory oversight is mandatory in a liberalized market. Without an efficient regulatory body, privatization will likely lead to the substitution of a public monopoly by a private one. Thus, the Ethiopian government must establish an independent and efficient regulatory body as soon as possible. Empowering the regulator by forming an independent board of directors, providing a reliable source of income, and running the regulator with a clear legal mandate is crucial in this regard.

Due to the existing large-scale limits of network coverage and gaps in the middle-mile and first-mile infrastructure, the Ethiopian telecom industry needs massive investment. Narrowing the digital divide requires investment mobilization from various stakeholders. This includes; allocating public funds for underserved areas, attracting CDNs investment, and utilizing universal service funds and universal service obligations. Seeing the geographical location and population size of the country, Ethiopia must host large IXPs. The government shall put various incentives to catalyze and utilize this untapped potential. Since lack of digital skills is the other hindrance, citizens’ digital literacy must be improved. Furthermore, local technical capacity should be strengthened to identify the main gaps in broadband infrastructure, develop applications and services, and avoid vendor-driven telecom techno-economic analysis in future projects. Access to locally relevant content is crucial as well. In this regard, the operator and Ethiopian government shall promote local content development and local hosting through various initiatives.

Finally, it is significant to highlight the relevancy of this paper for the various stakeholders. A lot should not be anticipated from this paper as it is merely a scoping study. However, with an additional detailed analysis, this work may be used as a foundation in drawing the Ethiopian telecom policies, strategies, and action-oriented concrete broadband roadmap. From this paper, stakeholders will likely get a snapshot picture of the causes for Ethiopia's poor telecom industry and pick up tips towards ensuring meaningful connectivity. It is also expected research and academic institutions, as well as the private sector, use this paper as a basis for a comprehensive study of Ethiopian telecom industry.

## Declarations

### Author contribution statement

Berhan Oumer Adame: Conceived and designed the experiments; Performed the experiments; Analyzed and interpreted the data; Contributed reagents, materials, analysis tools or data; Wrote the paper.

### Funding statement

This research did not receive any specific grant from funding agencies in the public, commercial, or not-for-profit sectors.

### Data availability statement

Data included in article/supp. material/referenced in article.

### Declaration of interests statement

The authors declare no conflict of interest.

### Additional information

No additional information is available for this paper.
